# Diagnostic and prognostic values of anti‐helicobacter pylori antibody combined with serum CA724, CA19‐9, and CEA for young patients with early gastric cancer

**DOI:** 10.1002/jcla.23268

**Published:** 2020-03-02

**Authors:** Xuyang Gong, Heng Zhang

**Affiliations:** ^1^ Department of Gastroenterology The Central Hospital of Wuhan Tongji Medical College Huazhong University of Science and Technology Wuhan China

**Keywords:** carbohydrate antigen, diagnosis, gastric cancer, helicobacter pylori, prognosis

## Abstract

**Background:**

To investigate the value of anti‐helicobacter pylori (Hp) antibody, serum carbohydrate antigen (CA)‐724, CA19‐9, and carcinoembryonic antigen (CEA) in the diagnosis and prognosis evaluation of young patients with early gastric cancer.

**Methods:**

A total of 200 young patients with gastric cancer from April 2014 to December 2015 were enrolled. A total of 206 patients with gastritis and 204 healthy subjects were also selected. Gastric cancer patients were followed up for 3 years, and the number of recurrences, metastasis, and death was recorded.

**Results:**

The positive rate of anti‐Hp antibody, CA724, CA19‐9, and CEA in young patients with early gastric cancer were significantly higher than those in gastritis and healthy subjects (*P* < .05), and was positively correlated with tumor stage, tumor size, and lymph node metastasis (*P* < .05). The predicting model was as follows: Logit (*P*) = 26.433‐3.014(CA724)‐3.908(CA19‐9)‐0.303(CEA)‐2.208(anti‐Hp antibody, Positive = 1; Negative = 0). This model had a high value in identifying young patients with early gastric cancer with AUC of 0.918, and the estimated probability was .806. Compared to patients with negative anti‐Hp antibody and low serum levels of CA724, CA19‐9, and CEA, the recurrence rate, metastasis rate, and mortality of patients with positive anti‐Hp antibody, high serum levels of CA724, CA19‐9, and CEA increased significantly (*P* < .05).

**Conclusion:**

This study indicated that anti‐Hp antibody combined with CA724, CA19‐9, and CEA had important value in the identification of young patients with early gastric cancer and were of great value in evaluating the risk of postoperative recurrence, metastasis, and death.

## 1 INTRODUCTION

Gastric cancer is a common malignant tumor, ranking fourth in the incidence of all global malignant tumors, and is a serious threat to people's health.^1^ There is obvious regional difference in gastric cancer incidence. It is much higher in Asia than that in Europe and America.^2^ According to prior reports, the incidence of gastric cancer in China ranked first of all malignant tumors.^3^ The incidence rate for men and women is about 2:1, and the mortality rate is the third‐highest of all malignant tumors.^3^ Although the incidence and mortality have been declining worldwide in recent years, the incidence in young population has not decreased yet. A recent study pointed out that the incidence of gastric cancer in young white people aged 25‐39 increased from 0.27/100 000 people in 2002 to 0.45/100 000 people in 2006, and the incidence increased by nearly 100%.^4^ The diagnosis of gastric cancer is not difficult, but the onset of gastric cancer is insidious. Most patients with early gastric cancer have no obvious symptom. Some patients have symptoms of nausea, vomiting, or similar upper gastrointestinal tract, which cannot be paid attention to. Therefore, the diagnosis rate of early gastric cancer is relatively low. With disease development, patient has obvious symptoms of upper gastrointestinal discomfort, such as abdominal pain, loss of appetite, weight loss, fatigue, nausea, vomiting, and suffocation. However, most patients had been in advanced stage when the above symptoms occurred. At this stage, the prognosis is poor because of the difficulty of treatment.

Patient's age of gastric cancer onset in China is mainly concentrated in middle‐aged and older people (ages older than 40 years).^5^ Although the incidence of gastric cancer is low in young people (age is equal to or less than 40 years old), it is gradually increasing in recent years because of the effects of industrialization, environmental pollution, and bad living habits (smoking, drinking, grilling or pickled foods, and irregular schedules, etc).^6^ A large number of clinical data and references showed that young gastric cancer had more specific clinical manifestations than middle‐aged gastric cancer, with a higher degree of malignancy, earlier metastasis, and poorer prognosis.^7^, ^8^ Studies had shown that the 5‐year survival rate of early gastric cancer surgery could reach more than 90%, while the 5‐year survival rate of postoperative gastric cancer is only 5%‐15%.^9^ In this case, it is worthwhile to improve the early diagnostic methods for young patients with early gastric cancer.

Anti‐helicobacter pylori (Hp) antibody, carbohydrate antigen (CA)‐724, CA19‐9, and carcinoembryonic antigen (CEA) are all markers for early gastric cancer screening.^8^, ^10^, ^11^, ^12^, ^13^ However, the relationship between their levels and the clinicopathological features of young patients with early gastric cancer, and the evaluation of postoperative recurrence, metastasis, and death have not been reported. This article selected healthy controls, patients with gastritis, and young patients with early gastric cancer, to explore the diagnostic and prognostic values of anti‐Hp antibody, CA724, CA19‐9, and CEA in young patients with early gastric cancer.

## 2 MATERIALS AND METHODS

### 2.1 Subject inclusion

A total of 200 young patients with gastric cancer diagnosed by pathology from April 2014 to December 2015 were enrolled. The patients were 23‐40 years old, and the average age was 32.17 ± 3.84 years. There were 122 males and 78 females. All patients were stage I‐II and were initially diagnosed. All patients were treated with chemoradiotherapy and excluded from other malignancies. Among them, there were 86 cases with tumor diameter ≥3 cm and 114 cases with <3 cm. The staging of all patients was based on a system developed by the American Cancer Association. A total of 206 patients with gastritis were also enrolled. The patients were 21‐39 years old, and the average age was 31.87 ± 4.02 years. There were 124 males and 82 females. The inclusion criteria for patients with gastritis: (a) The patient underwent gastroscopy and was diagnosed with gastritis; and (b) Patients have signed informed consent. Moreover, a total of 204 healthy subjects were selected in this study, aged 22‐40 years, with mean age of 32.65 ± 4.11 years, including 121 males and 83 females. The inclusion criteria for healthy subjects: (a) Subjects have signed informed consent; and (b) no underlying disease. There was no significant difference in age and gender among the three groups (*P* > .05), indicating they were comparable.

Exclusion criteria: (a) Patients had a history of other cancer diseases in the past; (b) patients with blood system disease such as iron deficiency anemia, aplastic anemia, megaloblastic anemia, and thrombotic thrombocytopenic purpura; (c) patients with acute blood loss, or a history of blood transfusion; (d) patients with pulmonary hypertension, acute coronary syndrome, ischemic stroke, and peripheral vascular disease (arteriosclerotic occlusive disease, arteriovenous thrombosis, aneurysm, etc), heart failure, atrial fibrillation, and hypertension, etc, and chronic vascular disease; (e) patients with acute and chronic infections caused by bacteria, fungi, and viruses; and (f) patients with autoimmune disease such as rheumatoid arthritis or systemic vasculitis.

### 2.2 Ethical approval statement

This study was approved by the Ethics Committee of The Central Hospital of Wuhan, Tongji Medical College, Huazhong University of Science and Technology (20140178), and the acquisition of specimens and clinical information was subject to oral or written informed consent obtained. Blood samples were collected in accordance with the Declaration of Helsinki.

### 2.3 Follow‐up of young patients with early gastric cancer

According to the time of patients included in the study, they were followed up for 36 months. The number of death and the corresponding death time during the follow‐up period were recorded. The follow‐up endpoint was defined as (a) death within 36 months; (b) withdrawal of the study within 36 months; and (c) follow‐up up to 36 months.

### 2.4 Observation and comparison indicators

The subjects fasted for more than 10 hours, and then, 10 mL of venous blood was taken by a professional medical staff using a blood vessel containing a coagulant. In addition, the patient's postoperative peripheral blood was also collected to assess the prognosis. Blood samples were centrifuged at 3600 *g* for 15 minutes at 4°C for 1 hour, and the upper serum was collected. CA724, CA19‐9, and CEA were detected by chemiluminescence immunoassay. The test instrument is the ARCHITECT i2000sr automatic immunoassay analyzer (Abbott Company), and the kit is the original imported test for it. The carbon‐13 (^13^C) urea breath test qualitatively determined the anti‐Hp antibody level. The subjects fasted for 12 hours. First, inject the first balloon and then take the ^13^C urea 1 capsule. After 30 minutes, the second balloon was blown out and tested. Experimental standard: super standard ≤4 is negative and >4 is positive.

### 2.5 Establishment of the logistic regression model

A logistic regression model for young patients with early gastric cancer was established. The formula is Logit (P) = *β*_0_ + *β*_1_ *X*_1_+*β*_2_ *X*_2_+…+*β*_n_ *X*_n_ = ln[*P*/(1‐*P*)], “*P*” means the probability of young patients with early gastric cancer, “*β*” means the coefficient of each parameter, and “*X*” means the value of each parameter.

### 2.6 Statistical analysis

All analyses were performed using SPSS 21.0 software. The measurement data were expressed as mean ± standard deviation (SD). Variance analysis and *t* test were used for comparison between multiple groups and two groups. The rate was compared using a chi‐square test. The Spearman method was used to analyze the correlation between each indicator and clinical features. The receiver operating characteristic curve (ROC) was used to analyze the diagnostic value of anti‐Hp antibody, CA724, CA19‐9, and CEA for young patients with gastric cancer. Survival curve analysis was performed using the Kaplan‐Meier method, and statistical difference between survival curves was calculated by log‐rank test. Logistic regression was used to calculate the odds ratio (OR) and 95% confidence interval (CI) for anti‐Hp antibody, CA724, CA19‐9, and CEA in predicting young patients with gastric cancer. The test level is *α* = .05.

## 3 RESULTS

### 3.1 Comparison of anti‐Hp antibody positive rate, CA724, CA19‐9, and CEA levels among three groups

The differences in anti‐Hp antibody positive rate and CA724, CA19‐9, and CEA levels among young patients with early gastric cancer and gastritis and healthy subjects were statistically significant (*P* < .05). The positive rate of anti‐Hp antibody and the serum levels of CA724, CA19‐9, and CEA in young patients with early gastric cancer were significantly higher than those in gastritis patients and healthy subjects (*P* < .05). Besides, the positive rate of anti‐Hp antibody in patients with gastritis was significantly higher than that in healthy subjects (*P* < .05), as shown in Table 1.

**Table 1:** Comparison of anti‐Hp antibody positive rate, CA724, CA19‐9, and CEA levels among three groups

Groups	Young patients with early gastric cancer (n = 200)	Gastritis (n = 206)	Healthy subjects (n = 204)	*F*/*χ^2^*	*P*
CA724 (U/mL)	2.36 ± 1.61	0.59 ± 0.29*	0.53 ± 0.34*	*F* = 5.675	.003
CA19‐9 (U/mL)	34.09 ± 15.24	16.32 ± 9.13*	15.87 ± 6.01*	*F* = 7.126	<.001
CEA (ng/mL)	1.65 ± 1.08	0.71 ± 0.33*	0.66 ± 0.40*	*F* = 4.009	.019
Anti‐Hp antibody					
Positive	156	96*	83*, ^#^	*χ^2^* = 65.482	<.001
Negative	44	110*	121*, ^#^		

Abbreviations: CA724, carbohydrate antigen‐724; CEA; carcinoembryonic antigen; CA19‐9, carbohydrate antigen 19‐9.

* Compared with gastric cancer, *P* < .05;

^#^ Compared with gastritis, *P* < .05.

### 3.2 Correlation between anti‐Hp antibody, CA724, CA19‐9, CEA, and clinicopathological features of young patients with early gastric cancer

Correlation analysis showed that anti‐Hp antibody positive rate and serum CA724, CA19‐9, and CEA levels had no significant correlation with age, gender, differentiation status, and tumor type of young patients of gastric cancer (*P* > .05, Tables 2 and 3, and Table S1). However, they did have significant positive correlations with tumor stage, tumor size, and lymph node metastasis (*P* < .05, Tables 2 and 3, and Table S1). Anti‐Hp antibody positive rate and serum CA724, CA19‐9, and CEA levels were all significantly increased in patients with tumors in stage II or tumor diameter ≥3 cm, and also patients with lymph node metastasis.

**Table 2:** Relationship between serum anti‐Hp antibody, CA724 level, and clinicopathological features of young patients with early gastric cancer

Clinicopathological features	Anti‐Hp antibody				CA724			
	Positive (n = 156)	Negative (n = 44)	*χ^2^*	*P*	High‐level group (n = 100)	Low‐level group (n = 100)	*χ^2^*	*P*
Age								
≥30	86	26	0.219	.640	53	59	0.731	.393
<30	70	18			47	41		
Gender								
Male	97	25	0.400	.527	58	64	0.757	.384
Female	59	19			42	36		
Differentiation status								
Well	74	17	1.072	.301	47	44	0.181	.670
Moderate‐poor	82	27			53	56		
Pathological type								
Papillary adenocarcinoma	79	21	1.014	.602	56	44	3.004	.223
Mucinous adenocarcinoma	35	13			22	26		
Tubular adenocarcinoma	42	10			22	30		
Tumor size								
≥3 cm	149	12	101.811	<.001	95	66	17.675	<.001
<3 cm	7	32			5	34		
Lymph node metastasis								
No	46	42	95.143	<.001	5	83	7.354	.007
Yes	110	2			95	17		
TNM staging								
I	47	40	80.770	<.001	15	72	66.097	<.001
II	109	4			85	28		

Abbreviations: CA724, carbohydrate antigen‐724; CEA; carcinoembryonic antigen; CA19‐9, carbohydrate antigen 19‐9.

**Table 3:** Relationship between serum CA19‐9 and CEA levels and clinicopathological features of young patients with early gastric cancer

Clinicopathological features	CA19‐9				CEA			
	High‐level group (n = 100)	Low‐level group (n = 100)	*χ^2^*	*P*	High‐level group (n = 100)	Low‐level group (n = 100)	*χ^2^*	*P*
Age								
≥30	57	55	0.081	.776	60	52	1.299	.254
<30	43	45			40	48		
Gender								
Male	55	67	3.026	.082	57	65	1.345	.246
Female	45	33			43	35		
Differentiation status								
Well	49	42	0.988	.320	46	45	0.020	.887
Moderate‐poor	51	58			54	55		
Pathological type								
Papillary adenocarcinoma, n	52	48	0.936	.626	50	50	2.564	.277
Mucinous adenocarcinoma, n	25	23			28	20		
Tubular adenocarcinoma, n	23	29			22	30		
Tumor size								
≥3 cm	89	72	9.205	.002	94	67	23.220	<.001
<3 cm	11	28			6	33		
Lymph node metastasis								
No	15	73	68.263	<.001	19	69	50.731	<.001
Yes	85	27			81	31		
TNM staging								
I	12	75	80.745	<.001	16	71	61.540	<.001
II	88	25			84	29		

Abbreviations: CA724, carbohydrate antigen‐724; CEA; carcinoembryonic antigen; CA19‐9, carbohydrate antigen 19‐9.

### 3.3 Model establishment using anti‐Hp antibody, CA724, CA19‐9, and CEA for young patients with early gastric cancer

**Figure 1:**
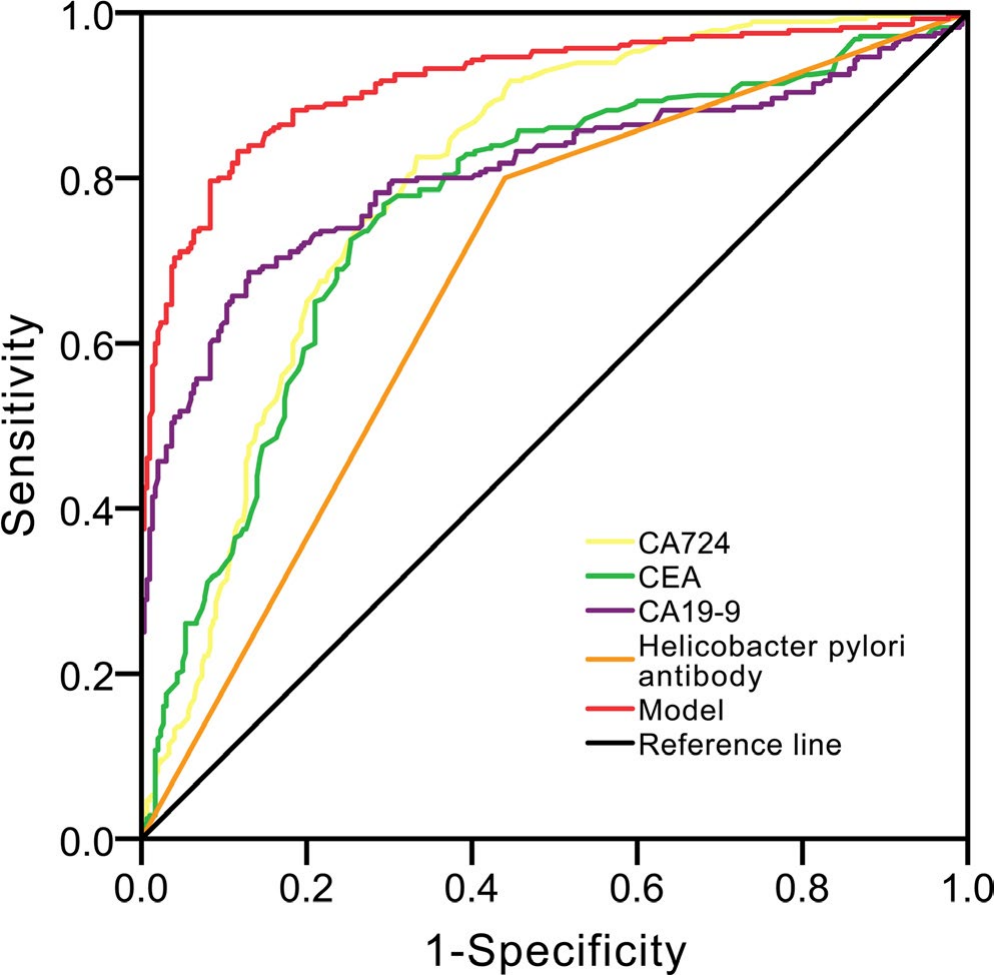
Receiver operating characteristic (ROC) curve analysis

ROC analysis showed that the area under curve (AUC) of anti‐Hp antibody, CA724, CA19‐9, and CEA in distinguishing young patients with early gastric cancer was 0.680, 0.798, 0.803, and 0.761, respectively (Figure 1 and Table S2). The abovementioned parameters were included in a logistic regression model. The final predicting model for patients with early gastric cancer prediction was as follows: Logit (P) = 26.433‐3.014(CA724)‐3.908(CA19‐9)‐0.303(CEA)‐2.208(Anti‐Hp antibody, Positive = 1; Negative = 0). This model had a high value in identifying young patients with early gastric cancer with AUC of 0.918 (Figure 1 and Table S2), and the estimated probability was .806 that meant if the probability was lower than .806, patients would be classified as young patients with early gastric cancer.

### 3.4 Prognostic value of anti‐Hp antibody, CA724, CA19‐9, and CEA

**Figure 2:**
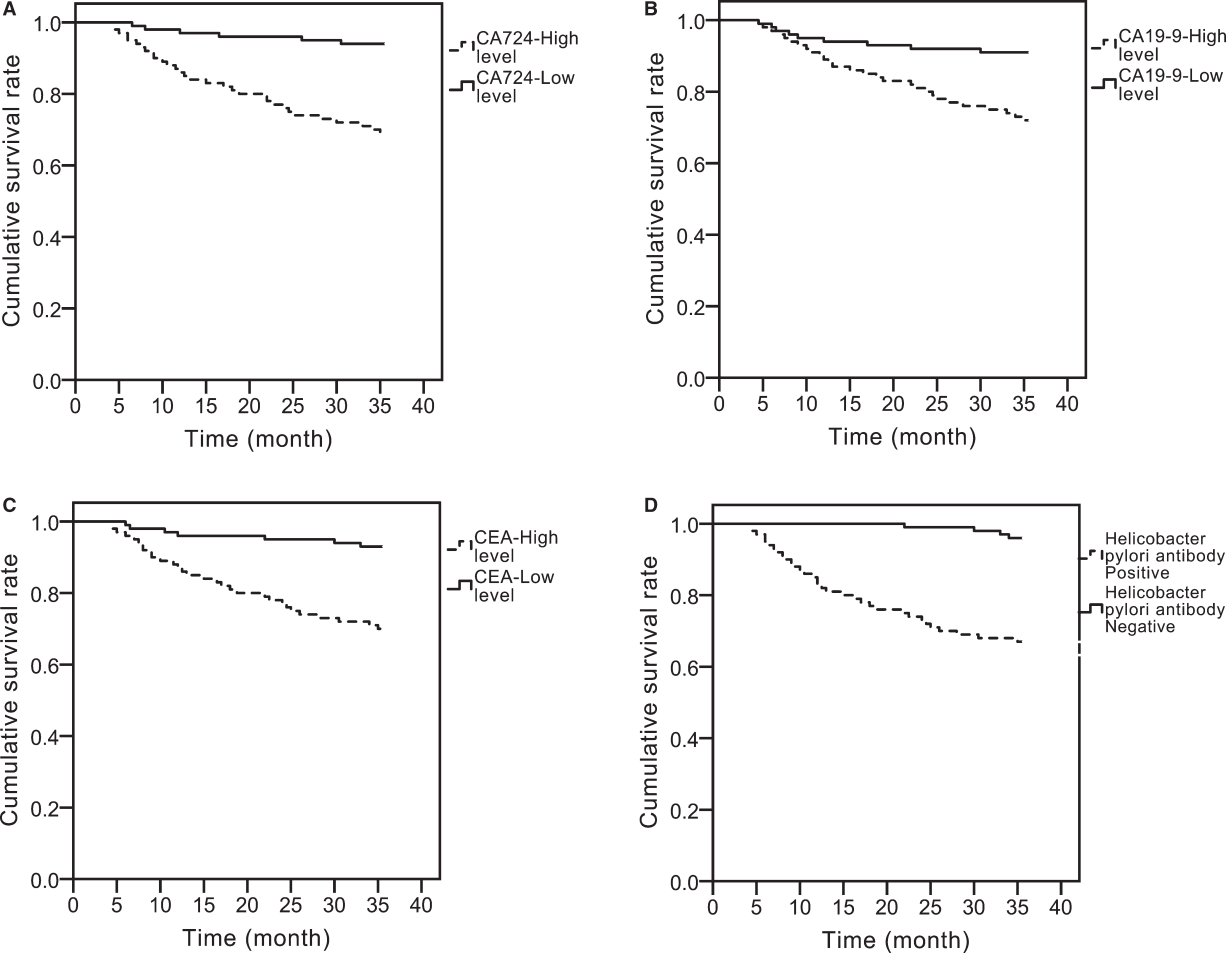
Prognostic value of the positive rate of anti‐Hp antibody, and levels of CA724, CA19‐9, and CEA in young patients with early gastric cancer. A, CA724. B, CA19‐9. C, CEA. D, Anti‐Hp antibody

Anti‐Hp antibody, serum CA724, CA19‐9, and CEA levels were detected in postoperative patients. According to the median serum levels of CA724, CA19‐9, and CEA (CA724: 1.36 U/mL; CA19‐9:24.19 U/mL; CEA: 1.38 ng/mL), patients were divided into the high‐ and the low‐level groups. Compared to patients with negative anti‐Hp antibody and low levels of CA724, CA19‐9, and CEA, the recurrence rate, metastasis rate, and mortality of patients with positive anti‐Hp antibody and high levels of CA724, CA19‐9, and CEA increased significantly (*P* < .05, Table 4 and Figure 2). Cox analysis showed that the positive anti‐Hp antibody and high serum levels of CA724, CA19‐9, and CEA were all risk factors for postoperative death in young gastric cancer patients (*P* < .05, Table 5).

**Table 4:** Analysis of prognostic value of positive rate of anti‐Hp antibody, CA724, CA19‐9, and CEA levels for recurrence, metastasis, and death

Groups	Recurrence	*χ^2^*/*P*	Metastasis	*χ^2^*/*P*	Death	*χ^2^*/*P*
CA724 (U/mL)						
High level (n = 100)	58	10.593/.001	76	56.186/.000	31	20.726/.000
Low level (n = 100)	17		23		6	
CA19‐9 (U/mL)						
High level (n = 100)	55	9.615/.002	81	79.388/.000	28	11.971/.001
Low level (n = 100)	20		18		9	
CEA (ng/mL)						
High level (n = 100)	53	9.058/.003	77	60.506/.000	30	17.543/.000
Low level (n = 100)	22		22		7	
Anti‐Hp antibody						
Positive (n = 156)	71	73.719/.000	89	42.195/.000	33	132.746/.000
Negative (n = 44)	4		10		4	

Abbreviations: CA724, carbohydrate antigen‐724; CEA; carcinoembryonic antigen; CA19‐9, carbohydrate antigen 19‐9.

**Table 5:** Cox proportional regression model analysis

Parameters	Partial regression coefficient	Standard error	Wald	*P*	HR	95%CI
Anti‐Hp antibody	1.671	.621	9.367	<.05	1.364	1.170‐1.877
CA724	2.329	.831	10.500	<.05	1.810	1.428‐2.379
CA19‐9	1.806	.701	7.879	<.05	1.702	1.371‐2.390
CEA	1.654	.567	4.655	<.05	1.321	1.124‐1.678

Abbreviations: CA724, carbohydrate antigen‐724; CEA; carcinoembryonic antigen; CA19‐9, carbohydrate antigen 19‐9.

## 4 DISCUSSION

In recent years, with the emphasis on gastric cancer and the adjustment of dietary structure, the incidence of this disease has decreased slightly overall, but the new cases have gradually shown a trend of youthfulness, which is a huge threat to human health. Since gastric cancer in young people has the characteristics of “fast progress and high degree of malignancy,” early clinical interventions are of particular importance. However, at present, there is still a lack of reports on postoperative monitoring and prognosis evaluation for young patients with early gastric cancer. In this study, we used the gastric cancer screening indicators, anti‐Hp antibody, CA724, CA19‐9, and CEA, to investigate the clinicopathological features of young patients with early gastric cancer, and to explore their predictive values in tumor recurrence, metastasis, and death of postoperative patients.

Our study found that the positive rate of anti‐Hp antibody and serum levels of CA724, CA19‐9, and CEA in young patients with early gastric cancer were significantly higher than those in patients with gastritis and healthy subjects, suggesting that the above four indicators might have the potential function to predict the progression of gastric cancer. Spearman's test showed that anti‐Hp antibody positive rate and serum levels of CA724, CA19‐9, and CEA had no significant correlation between age, gender, differentiation status, and tumor type of young patients with gastric cancer, but they did have significant correlations with tumor stage and tumor size. Patients in II stage, patients with tumor diameter ≥3 cm, and patients with lymph node metastasis trended to have higher positive rate of anti‐Hp antibody and higher serum levels of CA724, CA19‐9, and CEA. To further explore the prognostic value of the four indicators, we performed a 36‐month follow‐up of all patients. Compared with patients with negative anti‐Hp antibody and low serum levels of CA724, CA19‐9, and CEA, the recurrence rate, metastasis rate, and mortality of patients with positive anti‐Hp antibody and high serum levels of CA724, CA19‐9, and CEA significantly increased. Cox analysis showed that positive anti‐Hp antibody, high CA724, high CA19‐9, and high CEA levels were all risk factors for postoperative death in young gastric cancer patients. These results are different from the previous studies by Chae et al^14^ and Tas et al.^15^ Chae et al^14^ and Tas et al^15^ pointed out that the levels of markers such as CA724, CA19‐9, and CEA were not meaningful for the evaluation of the prognosis of patients with gastric cancer. We predict the reasons as follows: (a) The subjects we studied were young patients gastric cancer, while the average age of patients included in Chae et al^14^ was 61.1 ± 12.0 years, and the median age of patients included in Tas et al^15^ was 62 years. The age of patients in the above two studies was significantly older than that of patients in our study (32.17 ± 3.84 years old), which is the main reason contributed to the different results. (b) Young gastric cancer itself has rapid disease progression, and cancer cells have stronger ability to invade and metastasize.^7^, ^8^ This is different from the biological behavior of other types of gastric cancer, and this may be another important cause of different results.

However, this study has the following shortcomings: (a) This study is a retrospective study, the gastric cancer was confirmed before the study conducted, and some patients had already progressed to stage II as we all know that the different stage of the disease results in different prognosis and that may bias the results. Therefore, prospective studies with larger sample size are still needed to validate our results. (b) The underlying mechanisms of Hp, CA724, CA19‐9, and CEA in tumor progression of young gastric cancer remain to be further clarified. (c) The patients included in this study were from only one hospital, which caused case selection bias and affected the results. That means, our results are still waiting to be confirmed by multicenter cohort study.

In summary, we found that combining the anti‐Hp antibody with the serum CA724, CA19‐9, and CEA had important values in the identification of young patients with early gastric cancer and was of great value in evaluating the risk of postoperative recurrence, metastasis, and death.

## AUTHOR CONTRIBUTIONS

HZ researched literature and conceived the study. XG and HZ involved in protocol development, gaining ethical approval, patient recruitment, and data analysis. XG wrote the first draft of the study. All authors reviewed and edited the study and approved the final version of the study.

## ETHICAL APPROVAL

This study was approved by the Ethics Committee of The Central Hospital of Wuhan, Tongji Medical College, Hua Zhong University of Science and Technology (20140178).

## Supporting information

TableS1‐S2
