# Supplementary material for: Diagnostic and prognostic values of anti‐helicobacter pylori antibody combined with serum CA724, CA19‐9, and CEA for young patients with early gastric cancer
Source: J Clin Lab Anal. 2020 Mar 2;34(7):e23268. doi: 10.1002/jcla.23268 (PMC7370745; doi:10.1002/jcla.23268)
Supplement: Supplementary file 1 — TableS1‐S2 [file JCLA-34-e23268-s001.docx]

**Supplementary Table 1** Correlation coefficient between anti-Hp antibody positive rate, CA724, CA19-9, CEA levels and clinicopathological features of young patients with early gastric cancer

| Clinicopathological features | Anti-Hp antibody positive rate | | CA724 | | CA19-9 | | CEA | |
| --- | --- | --- | --- | --- | --- | --- | --- | --- |
|  | *r* | *P* | *r* | *P* | *r* | *P* | *r* | *P* |
| Age | 0.103 | 0.639 | 0.098 | 0.880 | 0.127 | 0.534 | 0.112 | 0.606 |
| Gender (Male) | 0.098 | 0.801 | 0.109 | 0.793 | 0.139 | 0.102 | 0.105 | 0.228 |
| Differentiation status (Well) | 0.098 | 0.624 | 0.116 | 0.520 | 0.154 | 0.097 | 0.136 | 0.209 |
| Pathological type | 0.063 | 0.901 | 0.122 | 0.431 | 0.108 | 0.117 | 0.164 | 0.086 |
| Tumor size (≥3cm) | 0.502 | 0.003 | 0.527 | 0.000 | 0.498 | 0.009 | 0.413 | 0.027 |
| Lymph node metastasis | 0.500 | 0.007 | 0.514 | 0.001 | 0.534 | 0.000 | 0.453 | 0.013 |
| TNM staging (II) | 0.499 | 0.002 | 0.503 | 0.001 | 0.587 | 0.001 | 0.487 | 0.004 |

Abbreviation: CA724, carbohydrate antigen-724; CA19-9, carbohydrate antigen 19-9; CEA; carcinoembryonic antigen

**Supplementary Table 2** The calculated performance values for different variables in distinguishing young patients with early gastric cancer

| Variable | AUC | 95% *CI* | *P* | Sensitivity (%) | Specificity (%) |
| --- | --- | --- | --- | --- | --- |
| CA724 | 0.798 | 0.762-0.834 | <0.001 | 82.5 | 66.7 |
| CA19-9 | 0.803 | 0.767-0.840 | <0.001 | 79.6 | 74.0 |
| Anti-Hp antibody | 0.680 | 0.636-0.724 | <0.001 | 78.0 | 56.3 |
| CEA | 0.761 | 0.721-0.801 | <0.001 | 77.1 | 69.7 |
| Model | 0.918 | 0.894-0.940 | <0.001 | 85.5 | 88.0 |

Abbreviation: CA724, carbohydrate antigen-724; CA19-9, carbohydrate antigen 19-9; CEA; carcinoembryonic antigen
